# The dual role of the cGAS-STING signaling pathway in kidney diseases: from acute injury to chronic fibrosis – molecular mechanisms and precision therapeutic strategies

**DOI:** 10.3389/fphar.2026.1882994

**Published:** 2026-08-05

**Authors:** Guangdong Qi, Fei Xue, Hualin Sun, Xinlei Yao, Qingyuan Liu

**Affiliations:** 1 Department of Endocrinology, Binhai County People’s Hospital, Yancheng, Jiangsu, China; 2 Jiangsu Key Laboratory of Tissue Engineering and Neuroregeneration, Key Laboratory of Neuroregeneration of Ministry of Education, Co-innovation Center of Neuroregeneration, Medical School of Nantong University, Nantong University, Nantong, Jiangsu, China

**Keywords:** acute kidney injury, cGAS-STING, chronic kidney disease, fibrosis, inflammation, mitochondrial DNA

## Abstract

The cyclic GMP-AMP synthase (cGAS)–stimulator of interferon genes (STING) signaling pathway, a central sensor of cytosolic DNA, plays a critical role in mediating innate immune responses. In recent years, research on this pathway in the field of kidney diseases has expanded explosively, extending from acute kidney injury (AKI) to various pathological conditions including chronic kidney disease (CKD), diabetic kidney disease (DKD), lupus nephritis (LN), and renal cell carcinoma (RCC). This review systematically summarizes the activation mechanisms and functional diversity of the cGAS-STING pathway in different kidney diseases. In AKI, mitochondrial DNA leakage, metabolic disturbances (lipid accumulation, lactate accumulation), and post-translational modifications (e.g., LDHB K156 lactylation) collectively activate this pathway, driving sterile inflammation. In CKD and renal fibrosis, the pathway promotes metabolic reprogramming, cellular senescence, and extracellular matrix deposition through canonical (TBK1–IRF3/NF-κB) and non-canonical (STING–PERK–eIF2α) signaling axes, as well as epitranscriptional regulation (e.g., METTL3-mediated m6A modification). In DKD and LN, its overactivation mediates podocyte injury and type I interferonopathy, respectively. Of note, in RCC, this pathway primarily exerts anti-tumor immune surveillance, highlighting its highly context-dependent functions. Although preclinical studies have demonstrated the therapeutic potential of various small-molecule inhibitors (e.g., RU.521, H-151) and natural product monomers or herbal formulas, clinical translation still faces four major challenges: the dual nature of pathway function (balancing host defense versus sterile inflammation), insufficient specificity and safety of existing inhibitors, lack of predictive biomarkers for therapeutic efficacy, and drug delivery difficulties arising from renal anatomical heterogeneity. To address these bottlenecks, this review proposes next-generation precision modulation strategies, including the development of tissue/cell-specific targeted delivery systems (e.g., biomimetic nanoscavengers), application of proteolysis-targeting chimera (PROTAC) technology, intervention in upstream metabolic and mitochondrial homeostasis, modulation of post-translational modifications, and combination therapies (e.g., with SGLT2 inhibitors, immune checkpoint inhibitors, or senolytics). Finally, we discuss key future directions in this field: advancing highly selective STING inhibitors/degraders into clinical trials, establishing combinatorial biomarker panels based on urinary mtDNA/cGAMP, and achieving precision medicine stratification based on patient-specific pathway activation subtypes. In conclusion, the cGAS-STING pathway has emerged as a central hub linking kidney injury to inflammation, metabolism, and fibrosis, and its precise modulation holds transformative therapeutic promise for hundreds of millions of patients with kidney diseases worldwide.

## 1 Introduction

Kidney diseases, whether manifested as acute kidney injury (AKI) with rapid onset or as chronic kidney disease (CKD) with a protracted course, have emerged as major global public health concerns (Wang K. et al., 2023). The high incidence, elevated mortality, and substantial socioeconomic burden imposed by end-stage renal disease (ESRD) render it imperative to elucidate the underlying pathogenic mechanisms and to develop novel therapeutic strategies (Skopelja-Gardner et al., 2022; Yamada and Yanagita, 2025). Although the etiologies of different kidney diseases vary considerably, sterile inflammation, metabolic dysregulation, mitochondrial dysfunction, and aberrant regulation of cell death pathways constitute their common pathophysiological foundations (Wang K. et al., 2023). In recent years, the innate immune system—particularly the discovery of cytosolic DNA sensing pathways—has provided a novel perspective for understanding these pathological processes (Poli et al., 2017). Consequently, a deeper dissection of the context-specific activation patterns of these pathways in distinct kidney diseases holds significant scientific value for the development of targeted interventions.

The signaling axis formed by cyclic GMP-AMP synthase (cGAS) and its downstream effector, stimulator of interferon genes (STING), serves as a core sentinel for cytosolic double-stranded DNA (dsDNA), triggering downstream inflammatory and type I interferon (IFN-I) responses (Skopelja-Gardner et al., 2022; Yamada and Yanagita, 2025; Liu et al., 2026; Jiang et al., 2026). However, its activation must be tightly controlled, as excessive or persistent signaling contributes to tissue pathology. This duality forms the central theme of this review. Of note, the function of the cGAS-STING pathway is highly dependent on cell type and disease stage: while its activation is essential for pathogen clearance during early infection (Gupta et al., 2026), chronic or excessive activation under sterile inflammatory conditions serves as a critical driver of tissue injury (Skopelja-Gardner et al., 2022). Therefore, precise modulation of the activity level of the cGAS-STING pathway represents a key prerequisite for achieving therapeutic benefits across different disease contexts.

In the healthy state, host-derived DNA is strictly compartmentalized within the nucleus and mitochondria, and the cGAS-STING pathway remains quiescent. However, in kidney diseases, various injurious stimuli—such as ischemia-reperfusion, drug toxicity, hyperglycemia, and immune complex deposition—can induce mitochondrial dysfunction and nuclear DNA damage. This leads to the leakage of self-DNA (particularly mitochondrial DNA, mtDNA) into the cytosol, where it is recognized by cGAS, thereby triggering a sterile inflammatory response (Yamada and Yanagita, 2025; Maekawa et al., 2019). Once dysregulated, this inherently defensive mechanism becomes a key driver of renal injury, inflammation, fibrosis, and chronic disease progression. Consequently, targeting the cGAS-STING activation cascade triggered by mtDNA leakage has emerged as a highly promising therapeutic strategy for kidney diseases.

Over the past 5 years, research on the role of the cGAS-STING pathway in kidney diseases has expanded explosively. Initial explorations in acute kidney injury (AKI) models have rapidly extended to CKD, diabetic kidney disease (DKD), lupus nephritis (LN), renal cell carcinoma (RCC), and even rare nephrotoxicity models (Jiao et al., 2025; Li et al., 2026; Feng et al., 2026). Furthermore, the scope of investigation has deepened from mere phenotypic descriptions to sophisticated molecular mechanisms, including metabolic reprogramming, epigenetic regulation, and organelle crosstalk. For instance, recent studies have revealed how specific metabolic enzymes—such as ACOT8 and PC—indirectly modulate the cGAS-STING pathway by regulating lipid metabolism or glycolysis (Zhang H. et al., 2026; Huang et al., 2025), and how non-histone lactylation (e.g., LDHB K156) serves as a bridge connecting metabolism and inflammation (Luo et al., 2026). In parallel, epigenetic regulation is increasingly recognized: METTL3-mediated m6A modification exacerbates renal inflammation and fibrosis by enhancing the stability of cGAS and STING1 mRNAs (Tsai et al., 2024). Moreover, a diverse array of intervention strategies targeting this pathway has emerged, ranging from chemically synthesized small-molecule inhibitors (e.g., RU.521, H-151) to traditional Chinese medicine monomers and formulas (e.g., sinomenine, astragalus polysaccharides, Qihuang Yishen Formula, and Compound Danshen Dripping Pills), as well as innovative nano-biomimetic delivery systems (e.g., neutrophil-mimicking nanoscavengers) (Zhang Z. et al., 2026; Jin et al., 2025; Guo et al., 2025; Shi et al., 2024). Although these varied interventions provide a wealth of candidates for clinical translation, they also underscore the critical need for precise selection based on the type of kidney disease.

However, despite encouraging preclinical evidence, the clinical translation of cGAS-STING inhibitors for the treatment of kidney diseases faces substantial challenges. These include the functional complexity of the pathway (protective during infection but detrimental in sterile inflammation), the potential risks of infection and malignancy associated with long-term inhibition, the lack of specific biomarkers to guide patient stratification, and the intrinsic anatomical and physiological heterogeneity of the kidney. Of particular concern is the essential role of the cGAS-STING pathway in defending against pathogenic infections—for example, the type I interferon response mediated by this pathway is critical for controlling leptospiral colonization in the kidney (Gupta et al., 2026). Consequently, systemic inhibition of this pathway may increase the risk of opportunistic infections. Moreover, the pathway may exert dual effects in renal cell carcinoma: on one hand, its activation can enhance antitumor immunity (Feng et al., 2026; Ji et al., 2021); on the other hand, a high cGAS-STING signature correlates with poor prognosis in patients with clear cell renal cell carcinoma (Wu et al., 2022), suggesting that caution is warranted when using STING inhibitors in individuals with tumors. Collectively, these challenges highlight that future research must focus on developing cell-type-specific delivery systems and identifying robust biomarkers capable of predicting therapeutic responses.

This review aims to systematically summarize the latest molecular mechanisms by which the cGAS-STING signaling pathway functions in various kidney diseases, objectively dissect the current bottlenecks and challenges in clinical translation, and propose future therapeutic strategies and research directions. Through this comprehensive analysis, we hope to provide an in-depth and integrated perspective for researchers and clinicians in nephrology, immunology, and drug discovery, thereby facilitating the transition from mechanistic studies to clinical applications. Ultimately, precise modulation of the cGAS-STING pathway holds promise as a key breakthrough that could transform the therapeutic landscape of kidney diseases.

## 2 The cGAS-STING signaling pathway

The core function of the cGAS-STING pathway is to serve as a sentinel for cytoplasmic DNA, monitoring potential danger signals within the cell. The cGAS protein contains a nucleotidyltransferase (Ntase) core domain and a C-terminal domain. In its ligand-free state, cGAS is maintained in an autoinhibited conformation to prevent aberrant responses to self-DNA. Upon binding to double-stranded DNA (dsDNA) longer than 45 base pairs, cGAS undergoes conformational changes, dimerizes or oligomerizes, and activates its catalytic site. It subsequently utilizes ATP and GTP as substrates to synthesize linear or cyclic dinucleotides; among these, 2‘3’-cGAMP is the most well-characterized product, which binds and activates STING with high affinity (Skopelja-Gardner et al., 2022). Of note, beyond its role as a classical DNA sensor, cGAS activity is tightly regulated by multiple factors. For instance, recent studies have identified that UNC93B1 (unc-93 homolog B1) negatively regulates the cGAS-STING signaling pathway by promoting autophagic-lysosomal degradation of STING, thereby preventing its overactivation (Zhu et al., 2022). Collectively, these observations indicate that a sophisticated homeostatic regulatory network exists within cells to precisely control the activation threshold of this pathway, ensuring appropriate immune responses.

STING is a transmembrane protein localized to the endoplasmic reticulum, with a C-terminal cytoplasmic domain containing a ligand-binding domain (LBD). Binding of cGAMP induces STING trafficking from the endoplasmic reticulum to the Golgi apparatus, during which STING oligomerizes and recruits and activates TBK1. Activated TBK1 not only phosphorylates STING itself to stabilize the signaling complex but, more importantly, phosphorylates the transcription factor IRF3. Subsequently, phosphorylated IRF3 dimerizes and translocates into the nucleus, initiating the transcription of type I interferons (predominantly IFN-β) and interferon-stimulated genes (ISGs). Concurrently, the STING–TBK1 complex also activates the NF-κB pathway via the IκB kinase (IKK) complex, inducing the expression of pro-inflammatory cytokines such as interleukin-6 (IL-6) and tumor necrosis factor-α (TNF-α) (Skopelja-Gardner et al., 2022; Li et al., 2025). This TBK1-dependent dual transcriptional output mechanism establishes cGAS-STING as a critical bridge linking DNA damage to sterile inflammation, thereby dictating its complex downstream biological effects.

**FIGURE 1:**
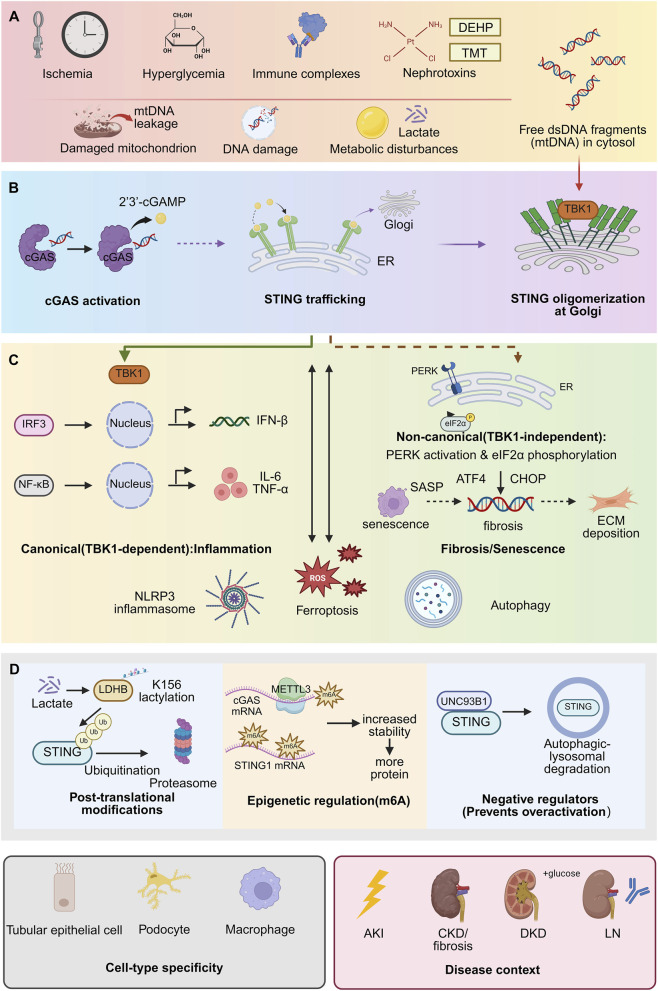
Four-layer overview of the cGAS-STING pathway in kidney diseases. **(A)** Injurious stimuli induce mitochondrial DNA (mtDNA) leakage into the cytosol. **(B)** cGAS synthesizes 2′3′-cGAMP, which activates the STING-TBK1 axis. **(C)** Canonical TBK1-IRF3/NF-κB signaling drives type I interferon and pro-inflammatory cytokine production, whereas the non-canonical STING-PERK-eIF2α axis promotes cellular senescence and organ fibrosis. **(D)** Pathway activity is fine-tuned by post-translational modifications (lactylation, ubiquitination), epitranscriptomic regulation (m^6^A), and the negative regulator UNC93B1. Key cell types and disease contexts are indicated. Created in BioRender.

In addition to the canonical TBK1-IRF3/NF-κB pathway, recent studies have identified multiple non-canonical pathways that greatly expand our understanding of the functional diversity of cGAS-STING. For instance, Zhang and colleagues discovered a STING-PERK-eIF2α axis that operates independently of TBK1-IRF3. Instead, this pathway directly binds and activates PERK (protein kinase R-like endoplasmic reticulum kinase), an endoplasmic reticulum stress sensor, thereby regulating cap-dependent mRNA translation. This mechanism plays a critical role in cellular senescence and organ fibrosis (Zhang et al., 2022). This finding explains why, under certain pathological conditions, STING activation can effectively drive disease progression even when type I interferon production is limited. Moreover, the cGAS-STING pathway exhibits extensive crosstalk with various forms of cell death, including pyroptosis and ferroptosis, as well as with metabolic reprogramming events such as glycolysis and lipid accumulation (Luo et al., 2026; Wang et al., 2026). For example, in renal ischemia-reperfusion injury, STING activation exacerbates kidney fibrosis by upregulating hexokinase 3 (HK3)-mediated lipid accumulation or by promoting PFKFB3-dependent glycolysis (Wang et al., 2026; Jiang et al., 2023). Collectively, the cGAS-STING pathway represents a highly complex and functionally versatile signaling network (Figure 1). Its context-specific activation patterns—varying across different cell types and pathological settings—determine vastly different biological outcomes, making it a promising yet challenging therapeutic target in the field of kidney diseases.

Beyond cGAS-STING, other cytosolic and endosomal nucleic acid sensors also contribute to renal innate immune signaling and frequently operate in parallel with this pathway. AIM2 (absent in melanoma 2) is a cytosolic dsDNA sensor that nucleates the AIM2 inflammasome, driving caspase-1-dependent IL-1β/IL-18 release and pyroptosis; in human CKD biopsies and in murine unilateral ureteral obstruction (UUO) models, AIM2 is upregulated in infiltrating macrophages and tubular epithelial cells, and Aim2-deficient mice show reduced renal injury, fibrosis, and inflammation (Komada et al., 2018). TLR9, an endosomal sensor of unmethylated CpG DNA (including mtDNA and NET-derived DNA), has similarly been implicated in renal injury, although its net effect on fibrosis appears to be model-dependent: TLR9 deficiency attenuates tubulointerstitial fibrosis after ischemia-reperfusion injury (Zheng et al., 2021), whereas TLR9 activation has paradoxically been reported to protect against fibrosis in the UUO model, suggesting context- or ligand-dependent effects that remain to be reconciled. Because AIM2, TLR9, and cGAS-STING can all be engaged by the same pool of self-DNA released during renal injury, these pathways are thought to act largely in parallel rather than in a strict hierarchy, and their combined or redundant activation may explain why selective inhibition of cGAS-STING alone sometimes yields incomplete protection. Dissecting the relative contribution and crosstalk of these DNA-sensing pathways in specific kidney disease contexts therefore represents an important avenue for future investigation.

## 3 Molecular mechanisms of cGAS–STING signaling in kidney diseases

Aberrant activation of the cGAS–STING pathway has been implicated in nearly all major categories of kidney disease, ranging from acute injury and chronic fibrosis to autoimmunity and malignancy (Table 1).

**TABLE 1:** Genetic or pharmacological inhibition of cGAS/STING ameliorates renal injury across kidney disease models.

Disease model	Cell type	Intervention	Effect on renal injury	PMID
Cisplatin-induced AKI	Tubular epithelial cells	STING genetic knockdown or pharmacological inhibition	Ameliorated renal injury	Maekawa et al. (2019)
Obstructive nephropathy (UUO)	Macrophages	cGAS^−/−^, STING^−/−^, or RU.521 (cGAS inhibitor)	Reduced macrophage activation, myofibroblast formation, and fibrosis	Jiao et al. (2025)
Renal fibrosis (PC-deficiency model)	Tubular epithelial cells	PC/SQOR axis restoration	Attenuated glycolysis and fibrosis	Huang et al. (2025)
Septic AKI	Renal tubular cells	LDHB K156 mutation	Alleviated kidney injury	Luo et al. (2026)
Septic AKI (NETs)	Macrophages	MD@NM neutrophil-mimetic nanoscavenger	Suppressed cGAS-STING signaling and promoted M2 shift	Zhang et al. (2026b)
Rhabdomyolysis-induced AKI	Macrophages	Astragalus polysaccharide	Attenuated renal damage via inhibition of M1 polarization	Sun et al. (2024)
DKD podocyte injury	Podocytes (*in vitro*)	STING genetic knockdown or pharmacological inhibition (STING or TBK1 inhibitor); not podocyte-specific knockout mice	Ameliorated proteinuria and podocyte injury	Zang et al. (2022)
DKD (db/db mice)	Systemic (whole-body)	STING whole-body knockout or nicotinamide riboside	Ameliorated albuminuria and renal inflammation	Myakala et al. (2023)
Kidney aging (aged mice)	Renal cells (*in vivo*)	STING inhibitor C-176 or pan-ERR agonist	Reversed cGAS/STING upregulation, albuminuria, and podocyte loss	Wang et al. (2023b)

### 3.1 AKI

AKI is characterized by a rapid decline in renal function, with ischemia–reperfusion injury (IRI), sepsis (SA-AKI), and drug toxicity (e.g., cisplatin) representing its three primary etiologies. In recent years, accumulating evidence has established that the cGAS–STING pathway plays a central pathogenic role across these distinct forms of AKI. Consequently, dissecting the precise mechanisms by which this pathway drives the initiation and progression of AKI has become a major focus of research in the field.

Mitochondrial DNA (mtDNA) leakage serves as a key upstream event that activates the cGAS–STING pathway. For instance, Maekawa and colleagues first demonstrated in a cisplatin-induced AKI model that mitochondrial damage leads to the release of mtDNA into the cytosol through BAX/BAK pores, thereby activating the cGAS–STING pathway and triggering inflammatory responses. Notably, genetic knockout of STING or pharmacological inhibition of STING significantly ameliorated renal injury (Maekawa et al., 2019). This mechanism has since been validated in various other AKI models. In septic AKI, Luo et al. reported that LPS stimulation induces glycolysis and lactate accumulation, subsequently promoting lactylation of the LDHB protein at lysine residue 156. Importantly, this modification acts as a critical molecular switch linking cGAS–STING-mediated metabolic reprogramming to NLRP3 inflammasome activation, and mutation of LDHB K156 markedly alleviated kidney injury (Luo et al., 2026). Furthermore, in a rhabdomyolysis-induced AKI model, astragalus polysaccharides were shown to attenuate renal damage by suppressing M1 macrophage polarization and the cGAS–STING pathway (Sun et al., 2024). Collectively, mtDNA leakage, as a common upstream event across multiple AKI subtypes, not only establishes the cGAS–STING pathway as a central mediator in AKI pathogenesis but also provides a strong rationale for targeting this pathway as a universal therapeutic strategy.

The interplay between metabolic disorders and cGAS-STING signaling has emerged as a research hotspot. On one hand, aberrant lipid metabolism can directly activate this pathway. Using multi-omics and Mendelian randomization analyses, Zhang et al. identified ACOT8 as a key pathogenic gene in renal ischemia-reperfusion injury (IRI). Specifically, ACOT8 is highly expressed in renal tubular epithelial cells, where it promotes the accumulation of palmitic acid, thereby altering the local lipid microenvironment. This subsequently activates the cGAS-STING pathway in macrophages, driving M1-type polarization and exacerbating inflammatory responses (Zhang H. et al., 2026). On the other hand, STING activation can reciprocally aggravate metabolic disturbances. Wang et al. demonstrated that in renal IRI and fibrosis models, STING activation upregulates hexokinase 3 (HK3) expression via NF-κB. The HK3-mediated lipid accumulation further promotes fibrotic progression, establishing a vicious cycle (Wang et al., 2026). Furthermore, in diabetic kidney disease, PCSK9 induces mitochondrial DNA damage and activates the cGAS-STING pathway, thereby triggering an inflammatory cascade (Feng et al., 2023). Collectively, these findings reveal a bidirectional regulatory relationship between metabolic reprogramming and cGAS-STING signaling, offering novel metabolism-targeted therapeutic strategies for AKI.

**FIGURE 2:**
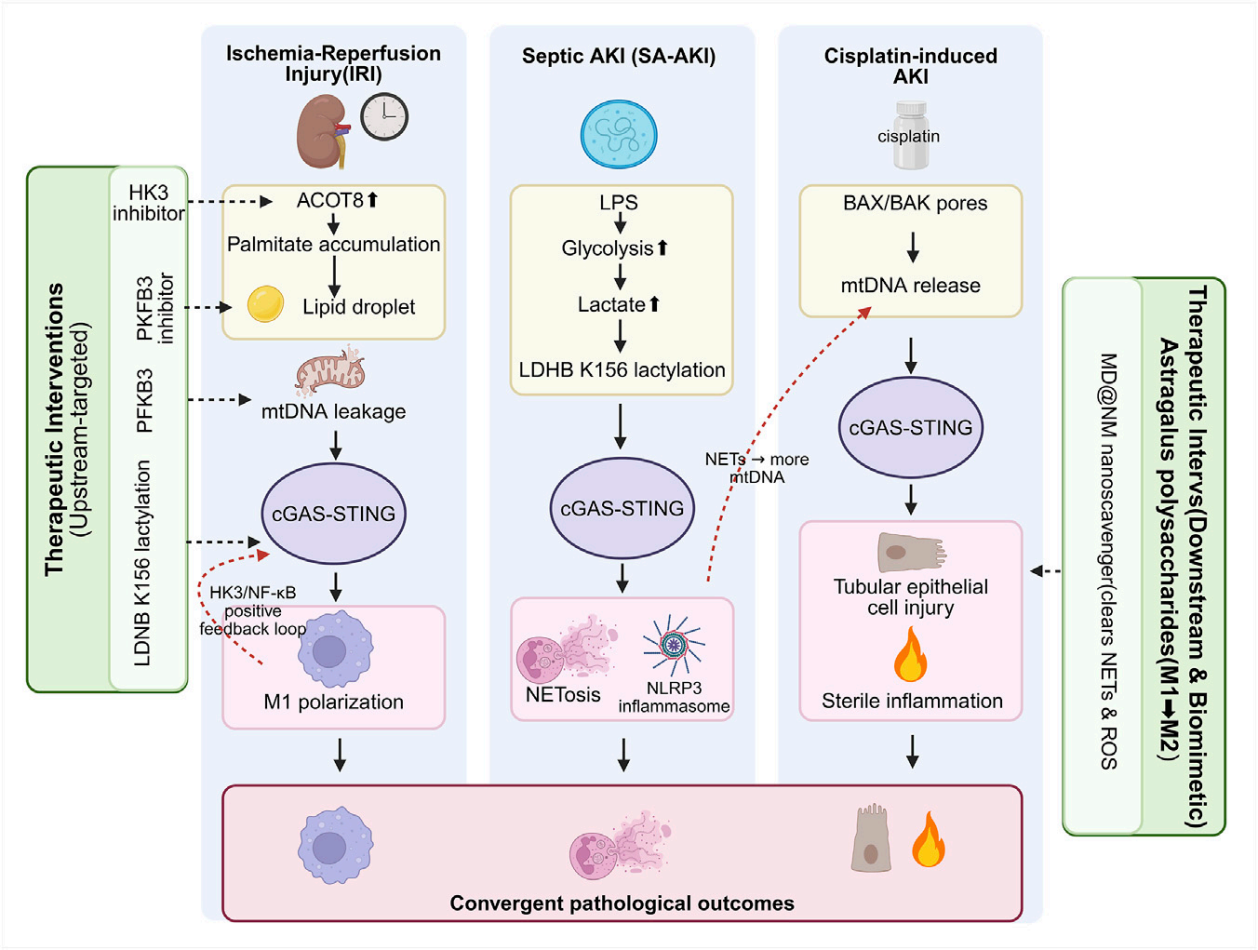
AKI-specific mechanisms of cGAS-STING activation. In ischemia-reperfusion injury (IRI), ACOT8-mediated palmitate accumulation drives M1 macrophage polarization. In septic AKI, LDHB K156 lactylation primes NLRP3 inflammasome activation and NETosis. Cisplatin triggers mtDNA release via BAX/BAK pores, leading to tubular injury. Therapeutic interventions include metabolic modulators, neutrophil-mimetic nanoscavengers (MD@NM), and *Astragalus polysaccharide*. Red loops highlight reciprocal amplification: NET-derived DNA fuels further mtDNA leakage, and the HK3/NF-κB axis exacerbates lipid accumulation. Created in BioRender.

The spatiotemporal dynamics of immune cells are critical determinants of AKI progression. Among these, macrophages serve as key effector cells of the cGAS-STING pathway in AKI. Beyond the aforementioned M1 polarization, loss of CX3CL1 has been shown to inhibit cGAS-STING activation by ameliorating mitochondrial dysfunction and reducing mtDNA leakage in macrophages, thereby promoting the transition toward an M2 reparative phenotype (Gong et al., 2025). Additionally, interventions targeting neutrophil extracellular traps (NETs) hold promising therapeutic potential. Zhang et al. developed a biomimetic nanoscavenger (MD@NM) that mimics neutrophil membranes to target the inflammatory microenvironment. This nanoscavenger clears NETs and reactive oxygen species (ROS), subsequently inhibiting cGAS-STING signaling and inducing macrophage conversion to the M2 phenotype, thereby effectively treating septic AKI(16). Together, these studies indicate that precise modulation of cGAS-STING activity in distinct immune cell populations—particularly promoting the transition of macrophages toward a reparative phenotype—represents a key therapeutic strategy for AKI. Several caveats apply to the AKI evidence summarized above. Nearly all causal data come from rodent knockout or pharmacological inhibitor studies, while human evidence remains largely correlative. These distinctions should inform how future AKI-targeted interventions are designed and interpreted (Figure 2).

### 3.2 CKD and renal fibrosis

The final common pathway in the progression of CKD is renal fibrosis, which is primarily driven by persistent, low-grade sterile inflammation. As a key DNA sensor, the cGAS-STING pathway not only serves as a bridge in the transition from AKI to CKD but also plays a critical role in the chronic progression of CKD. Therefore, a deeper understanding of the precise mechanisms by which this pathway contributes to renal fibrosis holds significant promise for the development of novel anti-fibrotic therapies.

Genetic and pharmacological studies have provided direct evidence for the pathogenic role of the cGAS-STING pathway in renal fibrosis. In a landmark study, Jiao et al. demonstrated, using global cGAS knockout (cGAS (−/−)) and STING knockout (STING (−/−)) mice as well as the selective cGAS inhibitor RU.521, that cGAS-STING signaling is a critical regulator of pro-inflammatory macrophage activation, myofibroblast formation, and renal fibrosis in an obstructive nephropathy model. Mechanistically, double-stranded DNA (dsDNA) released from injured tubular epithelial cells activated this pathway in macrophages, thereby amplifying the inflammatory response (Jiao et al., 2025). Furthermore, activation of this pathway within renal tubular epithelial cells directly promotes a pro-fibrotic phenotypic shift. For instance, Jiang et al. found that hypoxia activates the cGAS-STING-IRF3 axis to upregulate the expression of 6-phosphofructo-2-kinase/fructose-2,6-bisphosphatase 3 (PFKFB3), a key glycolytic enzyme, thereby driving a pro-fibrotic metabolic reprogramming in renal tubular epithelial cells (Jiang et al., 2023). Another study uncovered a protective role for the metabolic enzyme pyruvate carboxylase (PC): PC deficiency reduces its interaction with sulfide quinone oxidoreductase (SQOR), leading to SQOR degradation, which in turn triggers mitochondrial damage, mtDNA release, and cGAS-STING activation, ultimately exacerbating both glycolysis and fibrosis (Huang et al., 2025). Collectively, these findings from genetic and pharmacological studies firmly establish the central role of the cGAS-STING pathway in renal fibrosis.

Emerging research in epitranscriptomics has added a new dimension to our understanding of the mechanisms underlying upregulation of this pathway. Tsai and colleagues innovatively linked N6-methyladenosine (m6A) RNA methylation to cGAS-STING pathway activation. They found that the expression of the methyltransferase METTL3 is significantly elevated in fibrotic human and mouse kidneys, leading to increased m6A modification of cGAS and STING1 mRNAs. This modification enhances the stability of these mRNAs and upregulates their protein expression, thereby establishing a pro-fibrotic positive feedback loop. Notably, targeting METTL3 either genetically or pharmacologically effectively disrupted this loop and alleviated fibrosis (Tsai et al., 2024). Thus, targeting the epitranscriptional regulatory mechanism represented by METTL3 offers a novel intervention point for the treatment of CKD.

**FIGURE 3:**
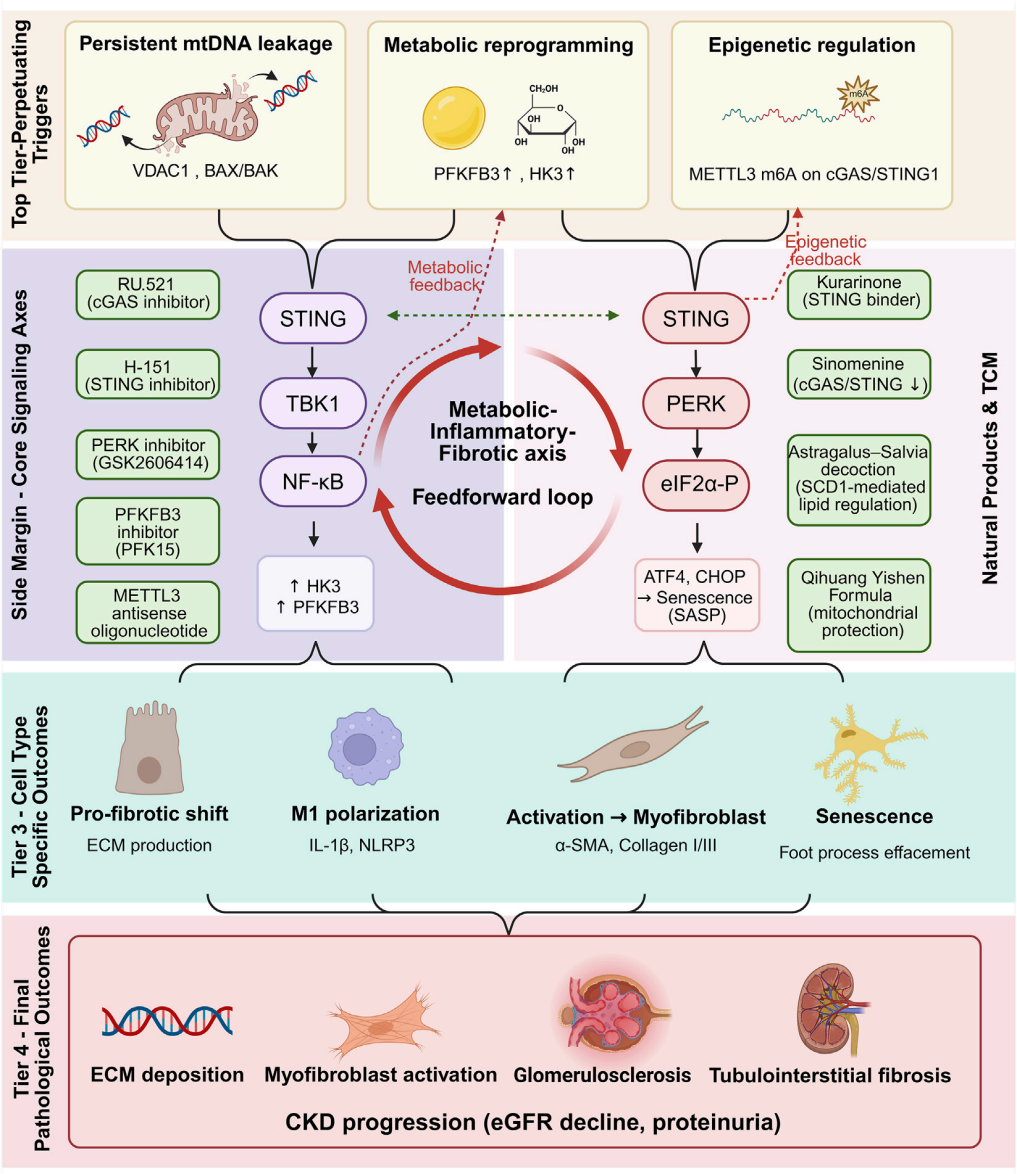
Chronic activation of the cGAS-STING pathway in CKD and renal fibrosis. Persistent triggers—including sustained mtDNA release, PFKFB3/HK3-driven metabolic reprogramming, and METTL3-mediated m^6^A mRNA methylation—feed into canonical (NF-κB) and non-canonical (PERK-eIF2α) signaling axes, which are linked by a central feedforward loop. Downstream consequences include pro-fibrotic transformation of tubular epithelial cells (TECs), M1 macrophage polarization, myofibroblast activation, and podocyte senescence, collectively promoting extracellular matrix (ECM) deposition and glomerulosclerosis. Potential intervention nodes are marked. Created in BioRender.

The crosstalk between endoplasmic reticulum (ER) stress and the non-canonical cGAS-STING pathway further enriches our understanding of the mechanisms underlying renal fibrosis. Beyond the classical TBK1-IRF3 axis, Zhang et al. identified a non-canonical STING-PERK-eIF2α pathway that plays a pivotal role in cellular senescence and organ fibrosis. This pathway operates independently of the classical unfolded protein response; instead, STING directly activates PERK kinase, initiating a translational program biased toward inflammation and cell survival. Notably, genetic or pharmacological inhibition of this non-canonical pathway significantly ameliorated both lung and kidney fibrosis (Zhang et al., 2022). Moreover, it has been proposed that the cGAS-STING pathway can exacerbate ER stress via the PERK pathway, with the two pathways forming a synergistic amplification loop that collectively drives the progression of kidney disease (Yamada and Yanagita, 2025). Taken together, these findings indicate that through complex crosstalk with pathways such as ER stress, the cGAS-STING pathway is deeply involved in the pathophysiology of CKD and renal fibrosis via multiple signaling axes. Several caveats apply to the CKD and fibrosis evidence summarized above. Much of the causal evidence derives from rodent models that track the transition from acute injury to fibrosis over a defined, relatively short timeframe, whereas human fibrotic kidney disease typically reflects years of cumulative, multifactorial injury; human data are largely limited to cross-sectional expression studies in biopsy specimens rather than longitudinal assessment of pathway activation. These distinctions have direct implications for biomarker selection and therapeutic timing in future anti-fibrotic trials (Figure 3).

### 3.3 DKD

DKD is a leading cause of CKD, characterized by a complex pathogenesis involving metabolic memory, lipotoxicity, oxidative stress, and chronic inflammation. Recent studies have demonstrated that the cGAS-STING pathway is robustly activated in response to these pathological stimuli and plays a critical role in DKD progression. Therefore, elucidating the precise mechanisms by which this pathway operates in DKD is essential for developing novel therapeutic strategies.

In the early stages of DKD, podocyte injury represents a key event driving disease progression. For instance, Zang and colleagues uncovered a distinct molecular mechanism underlying lipotoxicity-induced podocyte damage. In podocytes from diabetic and obese mice, lipotoxicity triggers mitochondrial damage, leading to the leakage of mtDNA into the cytosol via BAX pores, which subsequently activates the cGAS-STING pathway. Notably, the downstream signaling output of this pathway in this context is highly cell-type- and disease-context-specific, predominantly activating TBK1 and NF-κB rather than the canonical IRF3. Experimental evidence confirms that genetic STING knockdown in cultured podocytes, together with pharmacological inhibition of STING or TBK1, significantly ameliorates podocyte injury *in vitro* and, in the case of pharmacological STING inhibition, proteinuria in db/db mice *in vivo* (Zang et al., 2022). This finding underscores the divergence of STING downstream signaling across different cell types and disease contexts, providing a rationale for precision targeting of this pathway.

Furthermore, research by Khedr et al. has revealed age- and sex-dependent activation of the cGAS-STING pathway in DKD. They found significant sexual dimorphism in cGAS-STING pathway activation in aged T2DN rats, which may partially explain the sex differences observed in DKD progression (Khedr et al., 2024). In addition to lipotoxicity, other factors also contribute to pathway activation. For example, PCSK9 has been shown to exacerbate inflammatory responses in DKD by inducing mitochondrial DNA damage and activating the cGAS-STING pathway (Feng et al., 2023). Collectively, these findings outline a complex regulatory network of the cGAS-STING pathway in DKD, suggesting the possibility of targeting distinct upstream signals for therapeutic intervention. Consistent with this, Myakala and colleagues demonstrated that nicotinamide riboside suppressed cGAS/STING upregulation and, together with whole-body STING deletion, ameliorated albuminuria and renal inflammation in db/db diabetic mice (Myakala et al., 2023), providing direct genetic and pharmacological evidence that STING drives DKD progression.

In terms of therapeutic strategies, traditional Chinese medicine has shown considerable potential. Studies have found that the herbal formula Qihuang Yishen Formula (QHYS) alleviates renal tubular injury and inflammation in DKD by modulating the cGAS/STING pathway (Guo et al., 2025). Similarly, sinomenine has been demonstrated to attenuate renal inflammatory injury in db/db mice through inhibition of the cGAS-STING signaling pathway (Jin et al., 2025). In summary, the cGAS-STING pathway plays a central role in podocyte injury, inflammatory amplification, and disease progression in DKD. Targeting this pathway and its upstream and downstream key molecules offers new strategies and promise for DKD treatment. Future research should focus on developing more cell-specific regulatory approaches to achieve more precise and effective therapies for DKD. Several caveats apply to the DKD evidence summarized above. Podocyte-specific genetic knockout provides strong causal evidence for 1 cell type, but most other findings in this section derive from systemic pharmacological inhibition or global knockout models, leaving the relative contribution of tubular cells versus podocytes incompletely resolved. These distinctions should inform how future DKD-targeted interventions are designed and interpreted.

### 3.4 Lupus nephritis (LN)

LN is one of the most common and severe organ complications of systemic lupus erythematosus (SLE) and remains a major cause of progression to end-stage renal disease in SLE patients. As a prototypic autoimmune disease, SLE pathogenesis is closely linked to persistent overactivation of the type I interferon (IFN-I) signaling pathway. In this context, the cGAS-STING pathway—a critical DNA sensing and IFN-I-inducing cascade—plays a particularly prominent role in the onset and progression of LN.

Li and colleagues systematically reviewed the advances in understanding the cGAS-STING pathway in LN, highlighting that the levels of cGAS, STING, and IFN-I are significantly elevated in both SLE/LN mouse models and patient sera (Li et al., 2026). Mechanistic studies have revealed that, within the autoimmune milieu, immune complex deposition or mitochondrial DNA leakage can activate the cGAS-STING pathway in renal resident cells and infiltrating immune cells, thereby driving IFN-I responses and downstream inflammatory cytokine storms. Notably, genetic ablation of cGAS or STING substantially reduces autoantibody production and alleviates renal inflammatory injury, providing strong genetic evidence supporting the therapeutic targeting of this pathway in LN (Li et al., 2026). Although over 20 small-molecule inhibitors targeting cGAS or STING have been reported, clinical translation of these strategies remains challenging. These challenges include conflicting evidence from different mouse model studies, a lack of human clinical trial data for most candidate drugs, and the potential risk of immunosuppression following systemic inhibition of this pathway—which theoretically increases the likelihood of latent virus reactivation (Li et al., 2026). Therefore, in-depth dissection of the precise regulatory networks governing cGAS-STING activation in LN, along with the development of cell-type-specific or kidney-targeted inhibitors, represents a key direction for advancing clinical translation in this field. In summary, the cGAS-STING pathway occupies a central position in LN pathogenesis; although targeting this pathway holds considerable promise, future research must prioritize addressing the safety and efficacy issues that hinder its clinical translation. It should be noted that this evidence is drawn primarily from a narrative review synthesizing findings across SLE/LN mouse models and patient sera, rather than from kidney-specific genetic loss-of-function studies conducted within a dedicated LN model; the UUO-model evidence for cGAS-STING in renal fibrosis discussed in Section 3.2 is distinct and does not itself provide direct support for a kidney-intrinsic causal role in LN. The current evidence base should therefore be regarded as strongly suggestive rather than definitively causal, underscoring the need for kidney-specific interventional studies in *bona fide* LN models.

### 3.5 Renal cell carcinoma (RCC)

Unlike its pathogenic role in other kidney diseases, the cGAS-STING pathway in RCC primarily functions as a tumor suppressor and constitutes an essential component of immune surveillance. Specifically, Feng et al. demonstrated that GABA transaminase (ABAT) exerts tumor-suppressive effects in clear cell RCC (ccRCC). Mechanistically, ABAT interacts with protein arginine methyltransferase 5 (PRMT5) to enhance the activation of the cGAS-STING signaling pathway, thereby promoting IFN-β production and reducing regulatory T cell infiltration within the tumor microenvironment (Feng et al., 2026). Furthermore, activation of the cGAS-STING pathway via DNA damage response strategies, such as PARP inhibitor treatment, has been shown to enhance the therapeutic efficacy of CAR-T cells against RCC, offering a novel approach to overcome the tumor microenvironment barriers that limit immunotherapy in solid tumors (Ji et al., 2021). Nevertheless, the prognostic implications of this pathway are notably complex. Pan-cancer analyses reveal that cGAS-STING scores are elevated in kidney renal clear cell carcinoma (KIRC) and kidney renal papillary cell carcinoma (KIRP), where they correlate with poor prognosis. This paradox likely reflects that, although the pathway is activated in high-scoring tumors, these tumors have evolved mechanisms to evade or exploit the pathway-induced immunosuppressive microenvironment (Wu et al., 2022). Consequently, while the cGAS-STING pathway predominantly exerts anti-tumor immune effects in RCC, its high expression-associated poor prognosis underscores the existence of sophisticated immune evasion mechanisms in tumor cells. In summary, the cGAS-STING pathway plays a complex dual role in RCC, acting both as a critical effector of anti-tumor immunity and, when aberrantly activated, as a potential marker of tumor progression and unfavorable prognosis. Therefore, precise modulation of this pathway’s activity is paramount for developing effective immunotherapeutic strategies against RCC. It is important to distinguish the strength of evidence underlying these claims: the tumor-suppressive, mechanistically validated role of this pathway rests on defined genetic and pharmacological studies (Feng et al., 2026; Ji et al., 2021), whereas its association with poor prognosis is based on correlative pan-cancer transcriptomic analyses (Wu et al., 2022); these two lines of evidence address different questions and should not be conflated when interpreting the pathway’s net effect on RCC outcomes.

### 3.6 Other kidney-related diseases

**FIGURE 4:**
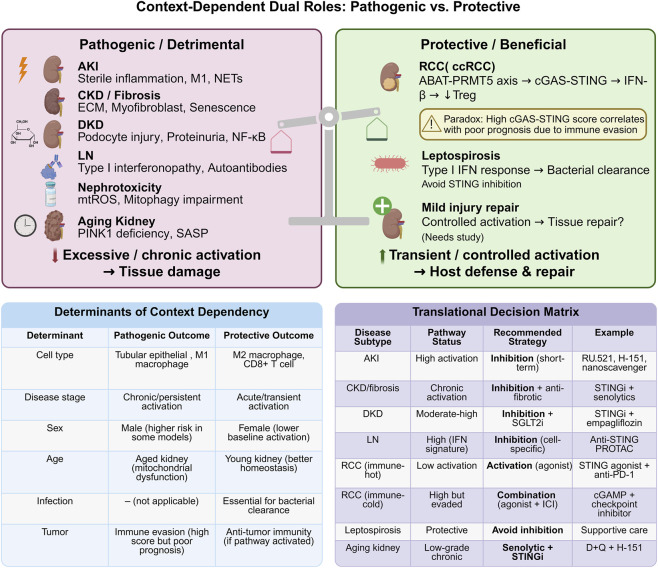
Context-dependent dual roles of the cGAS-STING pathway in kidney diseases. Left: Pathogenic contributions in acute kidney injury (AKI), chronic kidney disease (CKD), diabetic kidney disease (DKD), lupus nephritis (LN), nephrotoxicity, and kidney aging—primarily driving inflammation and fibrosis. Right: Protective functions in renal cell carcinoma (RCC) via anti-tumor immunity and in leptospirosis through bacterial clearance. A noted paradox: high cGAS-STING signature scores in RCC correlate with poor prognosis. Bottom tables summarize key determinants and a decision matrix for pathway inhibition versus activation. Created in BioRender.

Beyond the diseases discussed above, the cGAS-STING pathway is also implicated in additional kidney-related pathological contexts that further illustrate its dual, context-dependent nature. In cisplatin-induced acute kidney injury, targeted nanocarrier strategies have demonstrated therapeutic benefit: silk fibroin-delivered naringenin (Liu et al., 2023) and myricetin-loaded nanomicelles (Qi et al., 2023) both inhibit the mtDNA-cGAS-STING axis and alleviate nephrotoxicity, illustrating a nanodelivery-based intervention strategy for cisplatin nephrotoxicity distinct from the mechanisms discussed in Section 3.1. In the context of kidney aging, PINK1 deficiency exacerbates mitochondrial metabolic dysregulation and aggravates the senescence-associated secretory phenotype (SASP) and renal fibrosis through the cGAS-STING pathway (Ha et al., 2023), a finding reinforced by evidence that a pan-ERR agonist and, separately, the STING inhibitor C-176 each reverse age-related increases in cGAS/STING expression, albuminuria, and podocyte loss in aged mice (Wang XX. et al., 2023), together suggesting that suppressing this pathway may represent a strategy to delay kidney aging. Importantly, the role of the cGAS-STING pathway is not limited to sterile inflammation: during renal *Leptospira* colonization, the host relies on cGAS-STING-mediated type I interferon responses to clear the bacteria (Gupta et al., 2026), underscoring the pathway’s protective role in host defense and its complex dual nature across infectious and sterile inflammatory contexts. Together, these examples reinforce that the net effect of cGAS-STING activation in the kidney is highly context-dependent, ranging from pathogenic in nephrotoxic and aging-associated injury to protective during infection (Figure 4).

## 4 Challenges in clinical translation

Given the complex roles of the cGAS-STING pathway in kidney diseases, the clinical translation of its targeted therapies faces multiple challenges. This section systematically analyzes the core bottlenecks currently hindering progress, focusing on four aspects: the dual nature of the pathway, limitations of existing inhibitors, the lack of reliable biomarkers, and renal heterogeneity.

### 4.1 Challenges regarding inhibitor specificity, safety, and context-dependent risks

To date, although various small-molecule inhibitors targeting cGAS or STING have been reported, the vast majority remain at the preclinical stage, and their translational application in kidney diseases faces numerous challenges (Jiao et al., 2025; Li et al., 2026). For instance, RU.521 is a potent selective inhibitor of cGAS, whereas C-176/H-151 are commonly used small-molecule inhibitors of STING; both have shown therapeutic potential in several models of kidney disease (Jiao et al., 2025). Nevertheless, the clinical translational potential of these inhibitors remains limited, primarily due to the following issues. First, insufficient specificity is a major concern; some inhibitors may exhibit off-target effects, interfering with other innate immune signalling pathways (such as NF-κB or IRF3-independent pathways) and thereby eliciting unintended biological consequences. Second, their long-term safety profile remains unclear. Given that the cGAS-STING pathway plays essential roles in host defence, cellular homeostasis and tumour immune surveillance, chronic systemic inhibition of this pathway might increase the risk of opportunistic infections or impair the self-repair capacity of the kidney (Gupta et al., 2026). For example, cGAS-STING signalling confers protective effects against renal infection by certain pathogens (such as *Leptospira*), and its prolonged inhibition could increase the risk of renal colonization (Gupta et al., 2026). Third, unfavourable pharmacokinetic properties represent a critical obstacle limiting their *in vivo* application. Many inhibitors suffer from poor water solubility, low bioavailability and inadequate kidney-targeting ability, making it difficult to achieve effective drug concentrations at renal lesion sites. Furthermore, genetic polymorphisms in the STING protein itself mean that responses to STING agonists or antagonists may vary considerably among different populations, adding an extra layer of complexity to drug development and clinical application (Li et al., 2026). Therefore, a deep understanding of the limitations of current inhibitors and the development of next-generation molecules with improved specificity, safety and kidney-targeting capacity are essential to advance cGAS-STING-targeted therapies towards clinical translation. Beyond target specificity, long-term systemic inhibition of the pathway raises three further categories of concern that warrant deeper consideration. First, because cGAS-STING signaling is essential for host defense, chronic inhibition may impair antiviral and antibacterial responses; the requirement of cGAS-STING-mediated type I interferon signaling for controlling renal *Leptospira* colonization (Gupta et al., 2026) provides a concrete illustration of this risk. Second, in oncological contexts such as RCC, inhibition intended to reduce inflammation elsewhere in the body could theoretically impair the pathway’s anti-tumor immune-surveillance function (Feng et al., 2026; Ji et al., 2021), creating a potential trade-off between renal protection and tumor control that has not yet been systematically studied. Third, known species differences in STING biology, including differential sensitivity of human versus mouse STING to specific small-molecule agonists and antagonists, mean that pharmacokinetic and pharmacodynamic findings from rodent models cannot be assumed to extrapolate directly to humans; this caveat should be considered when prioritizing candidates for clinical development.

### 4.2 Lack of biomarkers for predicting and monitoring therapeutic efficacy

At present, the clinical diagnosis and progression assessment of kidney diseases rely primarily on non-specific indicators such as estimated glomerular filtration rate (eGFR) and urinary protein. However, these conventional parameters fail to reflect the specific activation status of the intrarenal cGAS-STING pathway, nor can they precisely distinguish inflammatory subtypes driven by this pathway. Consequently, for successful clinical trials of therapies targeting the cGAS-STING pathway, there is an urgent need to develop non-invasive or minimally invasive biomarkers that fulfill three key objectives (Wang K. et al., 2023): identifying patients with excessive cGAS-STING pathway activation to enable precise patient stratification (Skopelja-Gardner et al., 2022); dynamically monitoring target engagement and pharmacodynamic responses; and (Yamada and Yanagita, 2025) predicting long-term treatment responses and potential safety risks. Accumulating evidence suggests that urine represents an ideal source for discovering kidney-localized biomarkers. For instance, urinary levels of cell-free double-stranded DNA (dsDNA), mtDNA, and the second messenger 2′3′-cGAMP may directly reflect the activation status of the cGAS-STING pathway in renal tubular epithelial cells or infiltrating immune cells (Maekawa et al., 2019; Zang et al., 2022; Ma et al., 2022). Additionally, circulating levels of type I interferons (e.g., IFN-β) or specific interferon-stimulated gene (ISG) expression signatures could serve as indirect indicators of systemic immune responses. Of note, recent studies have also revealed that the myokine irisin not only exerts renoprotective effects but also exhibits a negative correlation with serum levels and the incidence of contrast-induced acute kidney injury (CI-AKI), suggesting its potential as a predictive biomarker for such injury (Peng L. et al., 2025). In summary, the development of a combinatorial biomarker panel based on urine and blood is critically important for advancing the clinical translation of cGAS-STING-targeted therapies. Addressing this biomarker gap will represent a pivotal breakthrough in moving the field from fundamental research toward clinical practice.

### 4.3 Renal heterogeneity and the challenge of drug delivery

The kidney is a highly heterogeneous organ, in which intricate structural units including the glomerulus, functionally segmented tubules, renal interstitium, and vasculature cooperate to perform finely tuned physiological functions. This diversity in structure and cell types implies that the key pathogenic cell types responsible for cGAS-STING pathway activation vary considerably across different kidney diseases. For instance, in DKD, podocytes appear to be the primary cell type affected by aberrant pathway activation (Zang et al., 2022). By contrast, in AKI and renal fibrosis, research has largely focused on tubular epithelial cells or infiltrating macrophages (Jiao et al., 2025; Zhang H. et al., 2026). Such cell-type specificity in pathological mechanisms poses a formidable challenge for pharmacological therapy: following systemic administration, it is difficult for drugs to achieve effective concentrations within specific pathogenic cell populations, which not only limits therapeutic efficacy but also increases the risk of systemic off-target effects. Consequently, the development of drug delivery systems capable of actively targeting specific renal cell types, for example, through ligand–receptor interactions or surface modification of nanocarriers, is of critical strategic importance for achieving precise intervention in the cGAS-STING pathway, enhancing therapeutic benefit, and minimizing off-target effects. Given the marked differences in cGAS-STING activation profiles among distinct renal cell subsets, the design of intervention strategies enabling cell-specific precision delivery has become a central breakthrough point for the clinical translation of this field.

## 5 Future therapeutic strategies

Although existing cGAS-STING pathway inhibitors have shown preliminary efficacy in preclinical models of kidney disease, their systemic inhibition may compromise immune surveillance and increase the risk of infections. Consequently, the field is transitioning from a paradigm of “complete blockade” toward “precision modulation”, aiming for spatiotemporally controlled and cell-selective pathway intervention to balance therapeutic benefits with safety. Based on this rationale, the following sections systematically describe multiple strategies for developing next-generation precision modulators.

### 5.1 Development of next-generation precision modulators

While various small-molecule inhibitors and natural products targeting the cGAS-STING signaling pathway have demonstrated therapeutic potential in kidney disease models, major challenges—including insufficient specificity, potential systemic immunosuppression, and target-independent side effects—remain obstacles to clinical translation. Accordingly, the development of next-generation modulators with enhanced precision has become a research priority. These innovative strategies aim to achieve tissue-specific, cell type–specific, and pathology-dependent pathway regulation.

#### 5.1.1 Tissue-specific or cell type–specific targeting

Tissue-specific or cell type–specific targeting represents a key strategy to improve the efficacy and reduce the off-target effects of cGAS-STING pathway inhibitors. Indeed, the cGAS-STING pathway exerts diverse pathophysiological functions across different renal cell types, including tubular epithelial cells, podocytes, macrophages, and endothelial cells (Li et al., 2025). For instance, proximal tubular epithelial cells are the primary sites of mtDNA leakage and cGAS-STING activation following ischemic, toxic, or septic injury (Zhang H. et al., 2026; Zhang Z. et al., 2026; Wang et al., 2026). Conversely, pathway activation in podocytes is closely associated with proteinuria in diabetic kidney disease (Zang et al., 2022). Therefore, delivering drugs specifically to the key pathological cell populations not only improves therapeutic efficacy but also avoids non-specific inhibition of physiological cGAS-STING functions in systemic compartments or other renal cell types. To achieve this goal, antibody–drug conjugates or aptamer-based targeted delivery systems can be employed—for example, conjugating cGAS/STING inhibitors to antibodies against kidney-specific cell surface markers, such as linking STING inhibitors to anti-megalin antibodies for renal tubule–targeted delivery. Moreover, a recent study provides an encouraging paradigm: neutrophil-mimicking nanoscavengers (MD@NM) leverage chemokine receptor–mediated active targeting to deliver DNase-1 and antioxidant nanozymes to injured kidneys, effectively clearing neutrophil extracellular traps (NETs) and reactive oxygen species (ROS), thereby suppressing excessive cGAS-STING activation (Zhang Z. et al., 2026). Collectively, such cell membrane–coated biomimetic nanoplatforms offer a valuable blueprint for kidney-specific immunomodulation and underscore the substantial promise of cell type–specific targeting strategies in treating renal diseases.

#### 5.1.2 Proteolysis-targeting chimera (PROTAC) technology

In contrast to small-molecule inhibitors, PROTACs leverage the ubiquitin–proteasome system to degrade the entire STING protein, thereby achieving more durable and complete inhibitory effects. This approach holds promise for overcoming resistance caused by protein mutations or feedback activation. Indeed, given that STING is consistently upregulated in various renal fibrosis and inflammation models (Jiao et al., 2025; Li et al., 2025; Wang et al., 2026), the development of STING-targeting PROTACs represents a highly promising future direction (Ding et al., 2020). This strategy not only effectively blocks both the scaffolding and downstream signaling functions of STING but may also fundamentally circumvent the compensatory pathway activation often associated with prolonged use of small-molecule inhibitors. Consequently, the development of efficient and kidney-specific STING-PROTACs will be a key breakthrough point for clinical translation in this field.

#### 5.1.3 Targeting upstream regulatory nodes

Rather than directly inhibiting cGAS or STING, an alternative strategy involves targeting their upstream triggers, such as mtDNA leakage, mitochondrial dysfunction, or metabolic dysregulation. In fact, this approach may suppress pathological inflammation while more selectively preserving the normal surveillance function of this pathway against low levels of DNA. Specifically, stabilizing mitochondrial membrane integrity, inhibiting voltage-dependent anion channel 1 (VDAC1) oligomerization, or clearing cytosolic mtDNA represent promising strategies worthy of in-depth exploration. For instance, inhibition of protein kinase C-δ (PKC-δ) has been shown to reduce VDAC1-mediated mtDNA release, thereby alleviating renal fibrosis (Wang et al., 2024). Moreover, CX3CL1 deficiency improves mitochondrial function and suppresses activation of the mtDNA–cGAS–STING pathway by stabilizing mitochondrial transcription factor A (TFAM) (Gong et al., 2025). Importantly, metabolic modulation—such as correcting acyl-CoA thioesterase 8 (ACOT8)-mediated palmitic acid accumulation (Zhang H. et al., 2026) or regulating glycolysis reprogramming through the pyruvate carboxylase (PC)/sulfide quinone oxidoreductase (SQOR) axis (Huang et al., 2025)—offers promising new avenues for upstream intervention. Collectively, indirect modulation of the cGAS–STING pathway through multidimensional interventions targeting mtDNA leakage into the cytosol and correcting metabolic disorders represents a more physiologically sound therapeutic direction for kidney diseases.

#### 5.1.4 Regulation of post-translational modifications

Post-translational modifications (PTMs) play a critical role in the activation and regulation of the cGAS-STING pathway. For instance, a recent study has revealed that lactylation of LDHB at lysine 156 (K156) couples cGAS-STING-mediated metabolic reprogramming with NLRP3 inflammasome activation in sepsis-associated acute kidney injury (SA-AKI) (Luo et al., 2026). Furthermore, METTL3-mediated m6A modification promotes sterile inflammation in renal fibrosis by enhancing the stability of cGAS and STING1 mRNAs (Tsai et al., 2024). Based on these findings, the development of specific inhibitory or mimetic small molecules, antibodies, or epitranscriptomic-based interventions—such as antisense oligonucleotides targeting METTL3 (Tsai et al., 2024) or pharmacological modulators of the EP300/HDAC2 lactylation axis (Luo et al., 2026)—may represent a highly specific therapeutic strategy. Collectively, the advancement of these next-generation precision modulation approaches holds promise for overcoming current translational bottlenecks in cGAS-STING-targeted therapies for kidney diseases, thereby offering safer and more effective personalized treatment options for patients. Therefore, in-depth dissection of the PTM regulatory network and the development of corresponding targeted intervention tools represent a key step toward translating cGAS-STING pathway research from bench to bedside in kidney disease clinical practice.

### 5.2 Nanotechnology-based targeted delivery systems

Given that most current small-molecule inhibitors of cGAS-STING suffer from poor solubility, low bioavailability and potential off-target toxicity, nanomedicine offers a powerful tool to overcome the challenges associated with renal drug delivery. It can significantly enhance the accumulation of inhibitors at kidney lesion sites while reducing systemic side effects (Zhang Z. et al., 2026; Xiao et al., 2025). Indeed, through sophisticated nanoscale design, multiple physiological barriers can be circumvented to achieve precise drug delivery. For example, passive targeting strategies exploit the intrinsic filtration properties of the kidney: nanoparticles with a hydrodynamic diameter of 10–50 nm can freely traverse the glomerular filtration barrier and undergo reabsorption in proximal tubules via receptors such as megalin, thereby enabling passive accumulation in renal tubular epithelial cells (Zhang Z. et al., 2026; Qi et al., 2023). This size-based passive targeting forms the basis for treating tubulointerstitial fibrosis and acute kidney injury. Building on this, active targeting strategies further enhance delivery precision by decorating nanoparticle surfaces with ligands or antibodies that specifically bind to receptors highly expressed on specific renal cell types—such as megalin, CXCR4 or integrin αV/β5 (Peng L. et al., 2025). For instance, conjugation with a CXCR4 antagonist allows selective delivery of STING inhibitors to injured tubular epithelial cells, thereby minimizing off-target effects.

Concurrently, biomimetic nano-delivery systems represent a cutting-edge direction in the field. As reported by Zhang and colleagues, a “neutrophil-mimetic nanoscavenger” (MD@NM) was constructed by coating DNase-1-loaded Mn_3_O_4_ nanozyme cores with neutrophil membranes (Zhang Z. et al., 2026). This biomimetic design not only achieves active chemotactic targeting via chemokine receptors such as CXCR2 expressed on the membrane, but also enables immune evasion through membrane proteins, thereby prolonging systemic circulation time. Moreover, this nanoscavenger effectively curbs excessive activation of the cGAS-STING pathway through synergistic scavenging of reactive oxygen species (ROS) and degradation of DNA derived from neutrophil extracellular traps (NETs) (Zhang Z. et al., 2026). Similar biomimetic strategies—for example, using platelet or macrophage membranes to coat nanoparticle cores—can also be harnessed to deliver STING inhibitors to inflamed kidney tissues, offering the potential for multi-mechanism synergistic therapy.

In summary, the design of intelligent nano-delivery systems that integrate passive targeting, active targeting and biomimetic functions represents a critical pathway to overcome renal drug delivery barriers and accelerate the clinical translation of cGAS-STING inhibitors. Therefore, by rationally combining these three strategies—leveraging size effects for basal accumulation, employing ligand–receptor interactions to enhance precision, and utilizing biomimetic membrane modifications to improve targeting and immune evasion—future nanoplatforms hold promise to markedly improve the pharmacokinetic profiles of inhibitors and reduce off-target toxicity, thereby opening new therapeutic avenues for kidney-related diseases.

### 5.3 Combination therapeutic strategies

Given the central role of the cGAS-STING signaling pathway in the progression of kidney diseases and its intricate crosstalk with multiple pathological pathways, 单一 targeting of this pathway may be insufficient to fully reverse established pathological changes. Consequently, the development of combination therapeutic strategies represents a crucial direction for optimizing clinical efficacy (Table 2).

**TABLE 2:** Combination therapeutic strategies integrating cGAS-STING pathway modulators with standard-of-care therapies in kidney diseases.

Combination strategy	Disease context	Proposed mechanism	PMID
SGLT2 inhibitors/ACEI-ARB + STING inhibitor	DKD (Conceptual proposal)	Concurrent correction of hemodynamic abnormalities and suppression of inflammatory signaling	Zhang et al. (2026c)
Astragalus polysaccharide (cGAS-STING inhibition)	Rhabdomyolysis-induced AKI	Inhibits M1 macrophage polarization and cGAS-STING pathway	Sun et al. (2024)
STING activation (e.g., Anlotinib) + immune checkpoint inhibitor (anti-PD-1/PD-L1)	RCC (mechanism shown in gastric cancer)	cGAS-STING/IFN-β activation converts immunologically “cold” tumors “hot”; not yet directly demonstrated in RCC	Yuan et al. (2022)
PARP inhibitor + CAR-T cell therapy	RCC	Activates cGAS-STING, promoting CAR-T infiltration and recruitment into tumor tissue	Ji et al. (2021)
NLRP3 co-blockade (via LDHB K156 axis)	Sepsis-associated AKI	Breaks the cGAS-STING–metabolic reprogramming–NLRP3 inflammatory amplification loop	Luo et al. (2026)
Dual cGAS/STING/TBK1 + TGF-β1/Smad blockade (Amlexanox)	Renal fibrosis (UUO model)	Concurrent anti-inflammatory and anti-fibrotic effects, attenuating ECM remodeling	Gairola and Kaundal (2025)
Huangqi-Danshen decoction (TCM) targeting SCD1	Renal fibrosis	Modulates lipid metabolism while inhibiting cGAS/STING signaling	Peng et al. (2025b)

#### 5.3.1 Combination with current standard of care

Integrating modulators of the cGAS-STING pathway with first-line clinical therapies holds promise for enhancing renoprotection through synergistic targeting of distinct pathogenic mechanisms. For instance, in patients with DKD, combining STING inhibitors with sodium–glucose cotransporter-2 (SGLT2) inhibitors or angiotensin-converting enzyme inhibitors/angiotensin receptor blockers (ACEI/ARB) may yield synergistic renoprotective effects by concurrently ameliorating hemodynamic abnormalities and suppressing inflammatory signaling cascades (Zhang XY. et al., 2026). Similarly, in a rhabdomyolysis-induced acute kidney injury (RIAKI) model, *Astragalus polysaccharide* has been shown to exert renal protection by inhibiting M1 macrophage polarization and the cGAS-STING pathway, suggesting its potential for combination with standard supportive care (Sun et al., 2024). Furthermore, for patients with renal cell carcinoma (RCC), combining STING agonists with immune checkpoint inhibitors (e.g., anti-PD-1 antibodies) may convert immunologically “cold” tumors into “hot” ones via activation of the cGAS-STING pathway, thereby improving immunotherapy response rates. In a different tumor type, Anlotinib was shown to enhance the antitumor efficacy of anti-PD-L1 therapy by activating the cGAS-STING/IFN-β pathway in gastric cancer cells (Yuan et al., 2022), suggesting a generalizable Anlotinib/cGAS-STING/IFN-β mechanism that has not yet been directly demonstrated in RCC. Moreover, PARP inhibitors can modulate the tumor microenvironment by activating the cGAS-STING pathway, thereby promoting CAR-T cell infiltration and recruitment into RCC tissues and enhancing the antitumor effect of CAR-T therapy (Ji et al., 2021). Collectively, combining cGAS-STING pathway modulators with current standard treatments not only offers novel strategies to enhance therapeutic efficacy and reduce toxicity but also warrants further investigation of such synergistic approaches in future clinical trials.

#### 5.3.2 Multi-pathway combinatorial blockade

Given the complexity of kidney diseases and the crosstalk among multiple pathogenic pathways, simultaneous blockade of the cGAS-STING pathway together with other key pathogenic pathways (such as the NLRP3 inflammasome and TGF-β/Smad) may yield more comprehensive therapeutic effects. Studies have shown that in sepsis-associated acute kidney injury (SA-AKI), activation of the cGAS-STING pathway promotes glycolytic reprogramming and lactate accumulation, which in turn facilitates NLRP3 inflammasome activation through K156 lactylation of LDHB, thereby forming a vicious cycle of “cGAS-STING–metabolic reprogramming–inflammatory amplification” (Luo et al., 2026). Similarly, in lipopolysaccharide (LPS)-induced AKI models, CX3CL1 deficiency suppresses cGAS-STING pathway activation by improving macrophage mitochondrial dynamics and reducing mtDNA leakage, consequently alleviating inflammatory responses (Gong et al., 2025). Collectively, these findings highlight the value of strategies that simultaneously target metabolic dysregulation and inflammatory pathways. Importantly, Amlexanox, by concurrently inhibiting cGAS/STING/TBK1 and TGF-β1/Smad signaling, exhibits enhanced anti-inflammatory and anti-fibrotic effects in a mouse model of unilateral ureteral obstruction (UUO), thereby effectively attenuating extracellular matrix remodeling (Gairola and Kaundal, 2025). Furthermore, traditional Chinese medicine formulas such as Huangqi-Danshen decoction alleviate renal fibrosis by targeting SCD1 to regulate lipid metabolism and inhibit cGAS/STING signaling, thus acting through the dual angles of metabolic remodeling and inflammatory control (Peng Y. et al., 2025). In summary, these studies collectively demonstrate that combinatorial blockade strategies targeting the cGAS-STING pathway—whether in combination with existing standard-of-care therapies or through simultaneous multi-pathway inhibition—represent a highly promising direction for improving therapeutic outcomes in kidney diseases and warrant further in-depth preclinical and clinical investigation.

### 5.4 Regulation of cellular senescence and the SASP

Cellular senescence is a critical driver of CKD progression, and excessive activation of the cGAS-STING pathway has been shown to regulate the translational program through non-canonical signaling axes, such as the STING–PERK–eIF2α pathway, thereby deeply participating in the senescence process and fibrogenesis (Zhang et al., 2022). Furthermore, activation of this pathway exacerbates the inflammatory microenvironment and impairs the transition of macrophages toward a reparative phenotype, further sustaining and amplifying the senescence-associated secretory phenotype (SASP) (Zhang Z. et al., 2026). Based on these insights, the use of senolytics (e.g., dasatinib plus quercetin) or senomorphics to eliminate senescent cells or suppress their SASP secretion may indirectly alleviate chronic cGAS-STING pathway stimulation and thereby retard kidney aging. Hence, future research should actively explore therapeutic strategies combining senolytics with specific STING inhibitors, with the goal of synergistically blocking the accumulation of senescent cells and their downstream inflammatory signals, thus providing more effective means to delay CKD progression.

In recent years, a growing body of research has revealed the substantial potential of traditional Chinese medicine (TCM) in modulating the cGAS-STING pathway to alleviate kidney injury. Indeed, numerous studies have demonstrated that various TCM monomers (e.g., icariin, kurarinone, sinomenine) and herbal formulas (e.g., Astragalus–Salvia decoction, Qihuang Yishen Formula) can effectively inhibit excessive cGAS-STING pathway activation, thereby exerting protective effects in multiple kidney disease models (Jin et al., 2025; Guo et al., 2025; Peng Y. et al., 2025; Liu et al., 2025). For example, the monomer kurarinone has been shown to directly target the STING protein, interfering with its interaction with IRF3 and consequently suppressing downstream inflammatory responses (Liu et al., 2025). Meanwhile, sinomenine alleviates inflammatory damage in diabetic kidney disease by inhibiting the expression of cGAS/STING and its downstream p-TBK1, p-IRF3, and NF-κB (Jin et al., 2025). Regarding herbal formulas, Qihuang Yishen Formula reduces renal tubular mitochondrial damage and mtDNA leakage via regulation of the cGAS/STING pathway, thereby mitigating inflammatory injury in diabetic kidneys (Guo et al., 2025). Additionally, Astragalus–Salvia decoction has been shown to inhibit the cGAS/STING signaling pathway by targeting SCD1 to modulate lipid composition, exerting anti-renal-fibrosis effects (Peng Y. et al., 2025). Clearly, these natural products and their derived formulations possess significant advantages, including multi-target effects and low toxicity, offering valuable resources for the discovery of novel lead compounds. Nevertheless, translating these TCM-derived achievements into clinically approved therapies still faces considerable challenges.

**FIGURE 5:**
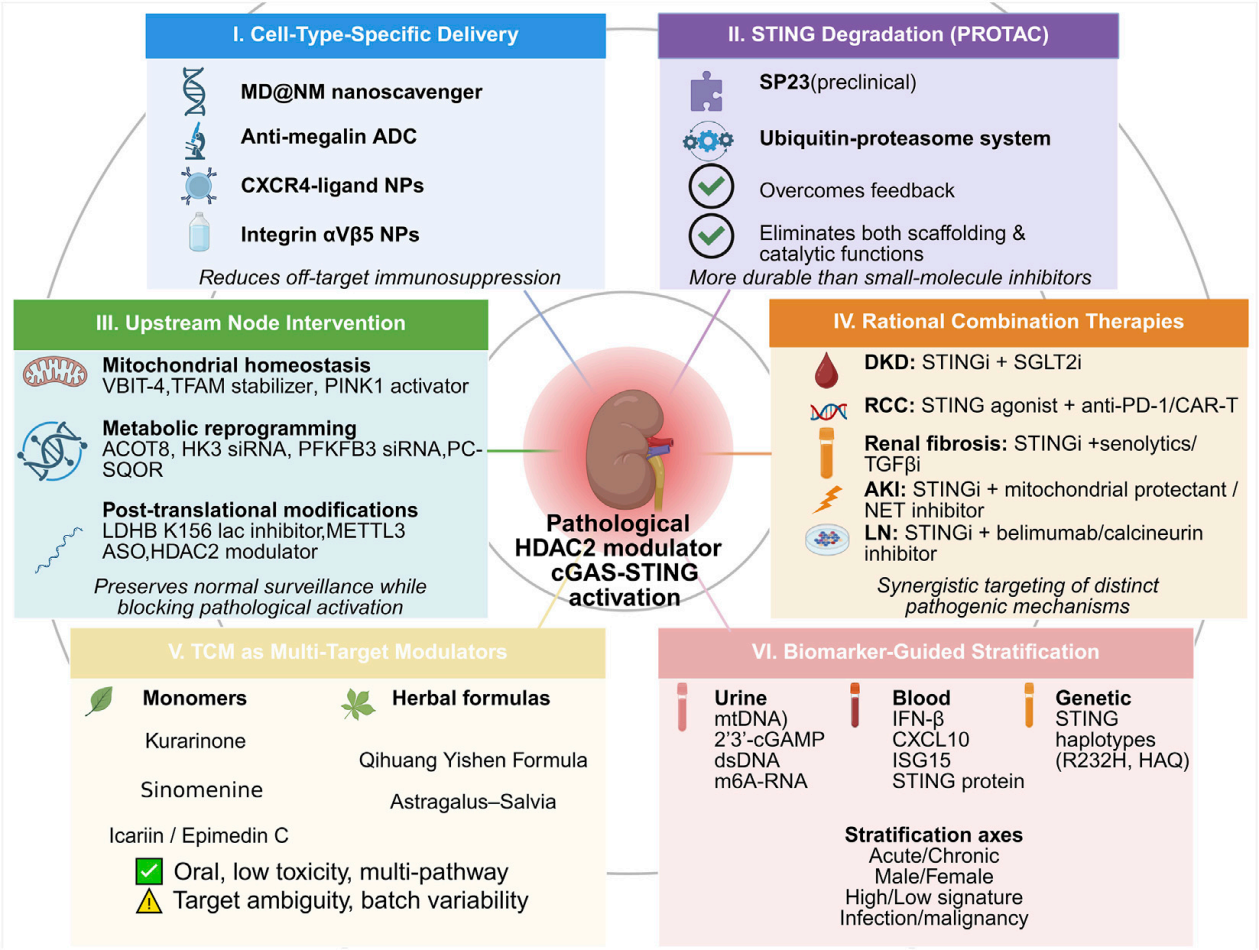
Six therapeutic modules for precision modulation of the cGAS-STING pathway. I: Cell-type-specific delivery systems (e.g., biomimetic nanoscavengers, antibody-drug conjugates). II: STING-targeting PROTACs for protein degradation. III: Upstream regulatory nodes (mitochondrial integrity, metabolic pathways, post-translational modifications). IV: Rational combination therapies (SGLT2 inhibitors, immune checkpoint inhibitors, senolytics). V: Traditional Chinese medicine modulators (kurarinone, sinomenine, herbal formulas). VI: Biomarker panels (urinary mtDNA/cGAMP, blood IFN-β, STING haplotypes). Created in BioRender.

Therefore, future research efforts should urgently focus on the following key directions. First, the direct targets should be identified: advanced techniques such as drug affinity responsive target stability (DARTS) and cellular thermal shift assay (CETSA) should be applied to systematically determine whether these natural products directly bind to cGAS or STING proteins, and to elucidate their specific binding sites and regulatory modes. Notably, using DARTS and CETSA, a recent study confirmed that Epimedin C, an analogue of icariin, directly binds to the STING protein and alters its stability (Lu et al., 2025). Second, the active components should be optimized: medicinal chemistry approaches should be employed to perform structural optimization and structure–activity relationship analyses of selected high-activity lead compounds, thereby improving their bioavailability, activity, and druggability while reducing potential off-target effects. Third, high-quality clinical studies should be conducted: rigorous and well-designed randomized controlled trials are needed to validate the actual efficacy, safety, and appropriate patient populations for single agents or formulas with well-defined mechanisms in various kidney disease patients, thus providing high-level evidence-based medical support for the modernization and internationalization of TCM. In summary, in-depth exploration and scientific optimization of traditional Chinese medicine resources promise to open novel and uniquely distinctive therapeutic strategies for kidney diseases by targeting the cGAS-STING pathway (Figure 5).

## 6 Conclusion and perspectives

In summary, the cGAS-STING signaling pathway has rapidly evolved from a fundamental immunological discovery into a central hub for understanding the pathogenesis of various kidney diseases. From mtDNA-triggered acute inflammation in AKI, to metabolic reprogramming-driven chronic fibrosis in CKD, and to type I interferonopathy in LN, this pathway is ubiquitously involved and plays dual or even multiple roles. A substantial body of preclinical evidence has painted a clear picture: precise modulation—rather than simply blanket inhibition—of cGAS-STING activity holds the key to treating kidney diseases.

Although the road to clinical translation is fraught with challenges—including the dual-edged nature of the pathway, limitations of current pharmacological agents, lack of reliable biomarkers, and the intrinsic complexity of the kidney—unprecedented opportunities also lie ahead. Next-generation precision modulators (e.g., PROTACs), smart nano-delivery systems, rational combination therapies, and deeper insights into aging and epigenetics collectively constitute powerful weapons for future breakthroughs. In particular, the rapid translation of novel mechanistic findings (e.g., post-translational modifications, metabolites, RNA modifications) into actionable intervention targets will greatly propel the field forward.

We anticipate that early-phase clinical evaluation of selective STING modulators, together with biomarker-guided patient stratification, represents a realistic near-to mid-term goal for acute kidney injury, diabetic kidney disease, and renal fibrosis, even though most next-generation modulators discussed in this review (e.g., PROTACs, biomimetic nanodelivery systems) and the biomarker panels proposed above remain at the preclinical or early validation stage and have not yet been tested in humans. Concurrently, precision medicine approaches based on patients’ genetic backgrounds, disease subtypes, and pathway activation status will require sustained translational investment before clinical realization. For nephrology, the cGAS-STING pathway is not merely a therapeutic target but also a beacon illuminating our understanding of organ-level sterile inflammation. Continued exploration and translation will undoubtedly bring new hope to hundreds of millions of kidney disease patients worldwide.
